# Mitochondrial DNA levels in Huntington disease leukocytes and dermal fibroblasts

**DOI:** 10.1007/s11011-017-0026-0

**Published:** 2017-05-16

**Authors:** Paulina Jędrak, Magdalena Krygier, Katarzyna Tońska, Małgorzata Drozd, Magdalena Kaliszewska, Ewa Bartnik, Witold Sołtan, Emilia J. Sitek, Anna Stanisławska-Sachadyn, Janusz Limon, Jarosław Sławek, Grzegorz Węgrzyn, Sylwia Barańska

**Affiliations:** 1 Department of Molecular Biology, University of Gdańsk, Wita Stwosza 59, 80-308 Gdańsk, Poland; 2 Department of Biology and Genetics, Medical University of Gdańsk, Gdańsk, Poland; 3 Institute of Genetics and Biotechnology, Faculty of Biology, University of Warsaw, Warsaw, Poland; 4 Polish Academy of Sciences, Institute of Biochemistry and Biophysics, Warsaw, Poland; 5 Department of Neurology, St. Adalbert Hospital, Copernicus PL Ltd., Gdańsk, Poland; 6 Department of Neurological and Psychiatric Nursing, Medical University of Gdańsk, Gdańsk, Poland; 7 Department of Bacterial Molecular Genetics, University of Gdańsk, Wita Stwosza 59, 80-308 Gdańsk, Poland

**Keywords:** Huntington disease, Mitochondrial DNA, Leukocytes, Dermal fibroblasts, Haplogroup

## Abstract

Huntington disease (HD) is an inherited neurodegenerative disorder caused by mutations in the huntingtin gene. Involvement of mitochondrial dysfunctions in, and especially influence of the level of mitochondrial DNA (mtDNA) on, development of this disease is unclear. Here, samples of blood from 84 HD patients and 79 controls, and dermal fibroblasts from 10 HD patients and 9 controls were analysed for mtDNA levels. Although the type of mitochondrial haplogroup had no influence on the mtDNA level, and there was no correlation between mtDNA level in leukocytes in HD patients and various parameters of HD severity, some considerable differences between HD patients and controls were identified. The average mtDNA/nDNA relative copy number was significantly higher in leukocytes, but lower in fibroblasts, of symptomatic HD patients relative to the control group. Moreover, HD women displayed higher mtDNA levels in leukocytes than HD men. Because this is the largest population analysed to date, these results might contribute to explanation of discrepancies between previously published studies concerning levels of mtDNA in cells of HD patients. We suggest that the size of the investigated population and type of cells from which DNA is isolated could significantly affect results of mtDNA copy number estimation in HD. Hence, these parameters should be taken into consideration in studies on mtDNA in HD, and perhaps also in other diseases where mitochondrial dysfunction occurs.

**Electronic supplementary material:**

The online version of this article (doi:10.1007/s11011-017-0026-0) contains supplementary material, which is available to authorized users.

## Introduction

Huntington disease (HD) is an autosomal, dominantly inherited neurodegenerative disorder, characterized clinically by involuntary movements with chorea as the most prominent symptom and psychiatric and cognitive symptoms. The underlying cause of the disease is the expansion of a CAG trinucleotide repeat in the huntingtin gene (HTT), resulting in an expanded polyglutamine (polyQ) stretch in the huntingtin protein (Htt). Mutant huntingtin (MHtt) undergoes intracellular aggregation in neurons and is probably responsible for the progressive degeneration of the brain (Gusella et al. 1983; MacDonald et al. 1993).

The most prominent pathological changes in HD affect the brain, especially the striatum. Of note, abnormalities are also observed in the peripheral tissues and lead to problems such as muscle atrophy, impaired glucose tolerance, testicular atrophy, weight loss and cardiac failure (Lanska et al. 1988; Browne et al. 1999; Stoy and McKay 2000; Van Raamsdonk et al. 2007; Turner et al. 2007). Although pathogenic mechanisms of HD are unclear, numerous abnormalities at the molecular level have been identified in the patients’ brains. These include increased lactate levels in the cortex and basal ganglia (which may reflect inefficient oxidative phosphorylation) (Jenkins et al. 1993), inhibition of mitochondrial complex II and III of the electron-transport chain in the brain (Gu et al. 1996), impairment of Ca^2+^ buffering capacity, and reduced mitochondrial ATP levels (Panov et al. 2002; Seong et al. 2005), induction of oxidative stress (Browne et al. 1997), and oxidative damage to DNA, both in the brain and other tissues. Most recently Yano et al. (2014) showed that MHtt directly inhibits the import of proteins involved in essential mitochondrial functions by binding to the mitochondrial import machinery. Such inhibition occurs in a tissue-, cell type– and time-dependent manner, and seems to be an early defect leading to further mitochondrial impairment.

One of the potential targets for pathological processes in HD is mitochondrial DNA (mtDNA). The mtDNA copy number is specific to the cell type and development stage, however, many other factors may influence the mtDNA level, e.g. energy requirements of the cell, the stage in the cell cycle, the redox balance in the cell and cell signaling processes (Michel et al. 2012). During embryonic development of animals, the mtDNA copy number is strictly controlled (Lefai et al. 2000). Mechanisms responsible for the regulation of the mtDNA level are still under debate, and it is still unclear when mtDNA molecules are replicated or degraded. Moreover, the question remains if observations made in other species hold true for human cells (Moraes 2001).

Altered mtDNA levels in different cells have been associated as a cause or consequence with a number of disorders, including multiple sclerosis (Blokhin et al. 2008), type 2 diabetes (Weng et al. 2009), biliary atresia (Tiao et al. 2007), neurodegenerative diseases, various cancers, e.g. renal cell carcinoma (Xing et al. 2008), breast cancer (Yu et al. 2007) and depression (Kim et al. 2011).

Results of various studies highlighted the role of altered mtDNA levels in HD and other poly-Q disorders, but with contradictory conclusions depending on the cell type. Chen et al. (2007) reported that the HD patients had a significantly increased amount of total mtDNA in leukocytes relative to control subjects. In contrast, a significant decrease of mtDNA was reported by Liu et al. (2008), in a group of HD patients compared to healthy subjects. A recently published report (Petersen et al. 2014) confirmed the latter observation about lower mtDNA copy number in HD patients relative to healthy controls.

HD phenotypes have been observed in leukocytes, including dysregulation of gene transcription (Borovecki et al. 2005; Anderson et al. 2008; Sassone et al. 2009; Kwan et al. 2012) and accumulation of MHtt (Weiss et al. 2012). We believed leukocytes to be good model cells since they can be taken from patients in a relatively simple way. Other tissues were rarely investigated, but a decreased amount of mtDNA in human HD skin fibroblast cell cultures, relative to the control fibroblasts, has also been reported (Siddiqui et al. 2012). Moreover, there are several reports indicating defects in such cells derived from HD patients, e.g. cell membrane disruption (Muratore 2013), decreased catalase activity (del Hoyo et al. 2006), impaired ganglioside metabolism (Maglione et al. 2010) or an increased level of MHtt in the cytoplasm (De Rooij et al. 1996). This indicates that dermal fibroblasts are also a good model for studies of HD. Therefore, the aim of this work was to assess differences between mtDNA levels in various cells (leukocytes and fibroblasts) in the largest group of HD subjects analysed to date, relative to controls, and to understand the reasons for the different results reported previously by various groups.

## Material and methods

### Study subjects

Leukocytes and dermal fibroblasts were analysed. Blood samples were withdrawn from 84 patients and 79 age- and sex-matched healthy subjects. HD patients were divided into two groups: 62 genetically confirmed HD patients with symptoms of the disease (symptomatic) and 22 genetically confirmed presymptomatic HD patients (at the preclinical stage of the disease). Skin biopsies were taken from two groups of subjects: 10 genetically confirmed HD patients with symptoms of the disease, and 9 age- and sex-matched healthy subjects. All skin biopsies were withdrawn from the skin on the forearm and immediately stored in physiological saline and transported to the laboratory, where biopsies were submerged in Hank’s Balanced Salt Solution (HBSS) supplemented with collagenase type II (200 units/ml, Life Technologies, Cat No 17102–015) and transferred to 37 °C for 18 h. Cells were dispersed, washed by centrifugation in HBSS, resuspended in cell culture medium and seeded on collagen-coated plates. Cells were routinely cultured in MEM supplemented with 10% FBS (PAN Biotech Germany).

**Table 1:** Basic characteristics of the study group (experiments with blood samples). NT not tested

Parameter	symptomatic HD patients’ values: range (average)		presymptomatic HD patients’ values: range (average)		healthy individuals’ values: range (average)	
	women	men	women	men	women	men
Number of subjects	37	25	17	5	52	27
Age (years)	31–75 (49)	33–77 (55)	22–57 (37)	19–39 (28)	24–68 (43)	27–86 (49)
CAG repeat expansion	36–55 (43)	38–53 (42)	38–55 (42)	39–44 (42)	NT	NT

All HD patients were recruited from REGISTRY-3 participants, examined at the Department of Neurology, St. Adalbert Hospital, Copernicus PL Ltd., Gdansk, Poland. REGISTRY-3 was an observational study of the European Huntington’s Disease Network (EHDN). The participants were questioned about their lifestyle (e.g. cigarette smoking, alcohol consumption, physical activity, current diseases and medications). Detailed family history was obtained from all patients and other factors that may influence the disease progression and severity, e.g. CAG repeats and age of onset (AOO) were considered. The severity of disease was assessed with the use of Unified Huntington’s Disease Rating Scale (UHDRS) by a certified neurologist. Control group subjects were selected carefully to avoid the influence of other diseases (like rheumatoid arthritis, diabetes, cancer) on the mtDNA level. All samples were collected at the Department of Neurology, St. Adalbert Hospital in Gdansk, Poland, from 2012 to 2014. Demographic and clinical summary of the studied groups is provided in Tables 1, 2 and 3. The study was approved by the local Ethics Committee of the Medical University of Gdansk (NKEBN/254/2011 and NKEBN/254–431/2012) and was conducted according to the tenets of the Helsinki Declaration. Written informed consents were obtained from all participants included in the study.

**Table 2:** Clinical data of the HD patients analysed in this study. **UHDRS**- Unified Huntington’s Disease Rating Scale, **HADS-SIS**- Hospital Anxiety and Depression Scale combined with the Snaith Irritability Scale, **SDMT** - Symbol Digit Modalities Test total correct, **MMSE** – Mini Mental State Examination. Scale ranges (normal to most severe) include total functional capacity (13–0), depression score from HADS-SIS (0–21), maximal chorea score (0–28), total motor score (0–124), oculomotor score (0–24), cognitive – SDMT - raw score (0–110), MMSE raw score (30–0)

Parameter		symptomatic HD patients’ values: range (average)	
		women	men
basic clinical data	age of onset (years)	19–67 (41)	31–64 (48)
	disease duration (years)	1–21 (8)	1–18 (9)
	time since motor onset (years)	0–17 (7)	1–18 (8)
motor function	UHDRS -total motor score	4–98 (55)	19–80 (52)
	UHDRS -maximal chorea score	2–26 (17)	4–27 (16)
	UHDRS -oculomotor score	2–18 (11)	6–14 (11)
functional capacity	The Total Functional Capacity (TFC) score	0–13 (7)	4–13 (8)
	UHDRS functional capacity	0–25 (18)	7–25 (18)
behavioral	Depression score from HADS-SIS	0–12 (6)	0–15 (7)
cognitive	MMSE raw score	11–30 (24)	16–30 (25)
	Cognitive – SDMT raw score	0–63 (23)	4–36 (18)
	Cognitive - semantic fluency raw score (animal names / min.)	0–27 (10)	4–21 (10)

**Table 3:** Characteristics of subjects whose skin biopses were investigated. NT not tested, NA not applicable

Parameter	symptomatic HD patients’ values: range (average)		healthy individuals’ values: range (average)	
	women	men	women	men
no. of subjects	4	6	4	5
Age (years)	40–64 (52)	41–65 (53)	41–56 (49)	43–68 (51)
CAG repeat expansion	36–43 (41)	39–43 (42)	NT	NT
Age of onset (years)	32–59 (47)	35–56 (46)	NA	NA
Disease duration (years)	4–8 (6)	3–13 (7)	NA	NA

### Blood sample collection and extraction of total DNA from leukocytes

Blood samples were drawn from the antecubital vein into two 4-ml, EDTA-containing tubes, from all subjects and delivered for isolation of DNA within 4 h. Each isolation was performed twice from two separate blood samples. Leukocytes were separated immediately from whole blood by LIZ-MIX (5 x LIZ-MIX for 1 l: 41.46 g NH_4_Cl; 2.3 g KHCO_3_; 10 ml 0.5 M EDTA). 4 ml of blood were centrifuged for 10 min at 1731 x g at 4 °C. The plasma was removed and 10 ml 1 x LIZ-MIX was added. The sample was gently mixed and placed on ice for 15 min to lyse erythrocytes. The sample was centrifuged for 10 min at 1731 x g at 4 °C. The supernatant was removed and the pellet was re-suspended in 10 ml of 1 x LIZ-MIX, and centrifuged under the same conditions as previously. Total cellular DNA was extracted up to 4 h after blood collection from a 200 μl leukocyte pellet suspended in PBS buffer using QIAamp® DNA Mini (QIAGEN), following the manufacturer’s protocol.

### Cell culture and total DNA isolation from fibroblasts

Fibroblasts were cultured in MEM (Biomed Lublin, Poland) supplemented with 10% FBS (PAN Biotech, Germany) and 1% antibiotic/antimycotic solution (Sigma-Aldrich, Germany) and maintained at 37 °C in a humidified atmosphere containing 5% CO_2_. DNA isolation was performed in the same way for all samples. Each isolation was performed twice from two separately grown cell cultures in passage 3–5. Total cellular DNA was extracted from approximately 5 × 10^6^ cells suspended in PBS buffer using QIAamp® DNA Mini (QIAGEN), following the manufacturer’s protocol.

### Measurement of leukocyte mtDNA content

Real-time quantitative PCR (qPCR) was used to determine the relative mtDNA copy number in leukocytes by using the estimation of threshold cycle (Ct) number of a nuclear, single copy gene, the beta-globin gene, and of a mitochondrial 16S rDNA fragment, in two independent runs. The method was carried out using LightCycler® 480 SYBR Green I Master (Roche) according to the manufacturer’s protocol. The qPCR was performed using primers and conditions shown in Supplementary Table S1. A total amount of either 10 ng or 5 ng DNA was used in each qPCR for determination of Ct for each gene. The efficiencies of qPCR were estimated during each run using serial dilutions of standard DNA fragments. The Ct values were accepted when the efficiency was between 90 and 105%. Each DNA sample was assayed in triplicate at two different amounts (10 ng and 5 ng), each 96-well plate was analysed twice for the same gene with DNA extracted from two biological repeats. On each plate, there was a negative control without DNA. The intra test was carried out using the same samples in a few runs to check reproducibility of the results in time.

### Haplogroup analysis

Haplogroup analysis was performed using a combination of Sanger sequencing and PCR-RFLP. The D-loop region was sequenced using primers D1, D2 and D3 according to (Taylor et al. 2001). Haplogroup affiliation was established on the basis of D-loop haplogroup markers described in MITOMAP combined with PhyloTree information. To establish haplogroup H and U affiliation, as there are no D-loop haplogroup markers, PCR-RFLP analysis was used as described by Torroni et al. (1996) and Piechota et al. (2004). PCR-RFLP analysis was also used when Sanger sequencing gave unreliable results.

### Common deletion analysis

The reactions were conducted as described previously (Soong and Arnheim 1996).

### Statistical analysis

The mtDNA copy number was calculated using the formula 2^ΔCt^ (ΔCt = Ct_(beta-globin)_-Ct_(16S)_) as the relative number of mtDNA to the nuclear DNA in leukocytes and fibroblasts. Normality of distribution was tested with the Shapiro-Wilk and K-S tests while homogeneity of variance was verified with the use of the Levene test. Statistical analyses were performed using Statistica 10.

Intergroup comparisons were performed with the use of two-way ANOVA as generalised linear model with LSmean statement and Tukey *post-hoc* test performed with SAS 9.3 programme (North Carolina, USA). mtDNA level was entered as a dependent variable and status of the analysed subjects (control, presymptomatic and symptomatic HD patients) and sex as a fixed/group factor. When 2 groups were compared (i.e. sex) *t-test* for independent samples or U Mann–Whitney test was used, depending on the data distribution and sample size. Since we have a small number of the cell lines besides the results of statistical tests crude data of mtDNA levels of particular individuals are presented.

Linear regression analysis was performed in the comparisons of mtDNA/nDNA relative copy number in leukocytes with age (years), disease duration (years), length of CAG expansion, the Total Functional Capacity (TFC) score, UHDRS functional, motor score, oculomotor score, maximal chorea score, depression score from HADS-SIS, time since motor symptoms onset, MMSE score, SDMT score and semantic fluency score between control group, presymptomatic HD patients and symptomatic HD patients with and without division into sexes (not all data were available for all of the study groups). In order to identify the potential association between symptom severity mentioned above with mtDNA level in leukocytes an analysis of covariance (ANCOVA) was carried out. Correlation, regression and ANOVA analyses were not performed unless clinical parameters/factors were assessed and available, or the number of analysed subjects was lower than 10. All analyses were performed with the alpha level set at 0.05.

## Results

### mtDNA/nDNA relative copy number is increased in HD leukocytes, but decreased in HD fibroblasts, relative to control cells

**Fig. 1:**
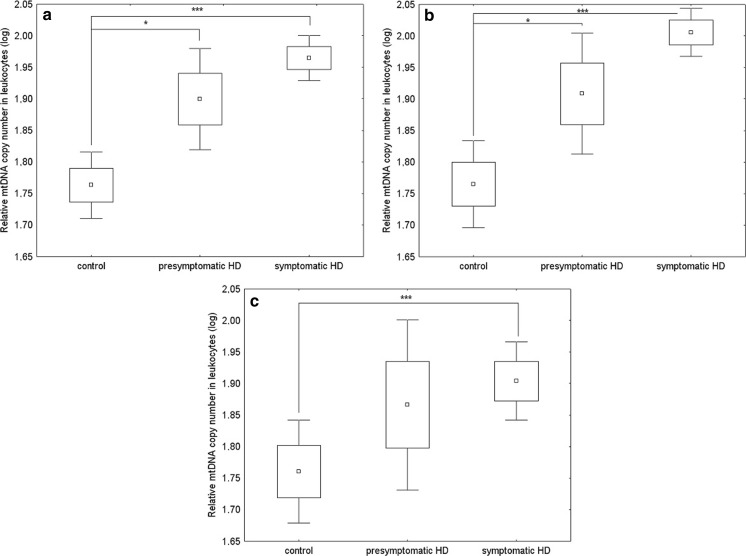
The relative mtDNA copy number in leukocytes of healthy subjects, presymptomatic and symptomatic HD patients without division into sexes (**a**), in women (**b**), and in men (**c**): *p < 0.05, ** *p* < 0.01, ***p < 0.001, using ANOVA on log-transformed data. The box and whisker plots represent average, average ± standard error, and average ± 1.96* standard error

In order to test mtDNA levels in cells of HD patients relative to control subjects, we isolated DNA from leukocytes and fibroblasts and determined mtDNA/nDNA ratios on the basis of real time PCR analysis. Our results showed a large variation in mtDNA/nDNA relative copy number in leukocytes and fibroblasts among individuals both among HD patients and controls (Fig. 1). This corroborated previous data indicating that the amount of mtDNA is specific to a person and depends on many individual factors, even among healthy subjects (Liu et al. 2008).

Raw data did not have a normal distribution thus they were log transformed. Such transformation changed the distribution to normal allowing us to use subsequent parametric statistical models. Since the research design was unbalanced (groups were not of equal sizes) results were analysed using two-way ANOVA LSMean. mtDNA level was entered as a dependent variable and status of the analysed subjects (control, presymptomatic and symptomatic HD patients) and sex as a fixed/group factor. This revealed a significant impact of disease status on mtDNA variability (*p* < 0.0001), when either symptomatic HD patients, presymptomatic and healthy subjects were analysed, but no impact of sex or sex-disease status interaction. The average mtDNA/nDNA relative copy number in leukocytes was significantly higher in symptomatic HD patients relative to the controls. Additionally, when analysis was restricted to symptomatic HD patients and healthy subjects, sex was found to impact mtDNA variability significantly (*p* < 0.05). Differences in mtDNA levels between women and men in symptomatic HD patients led us to analysis of differences using the t-student test which revealed significantly higher levels of mtDNA in women (*p* < 0.01). We did not observe significant differences in mtDNA copy number between presymptomatic and symptomatic HD patients (with and without division into sexes).

**Fig. 2:**
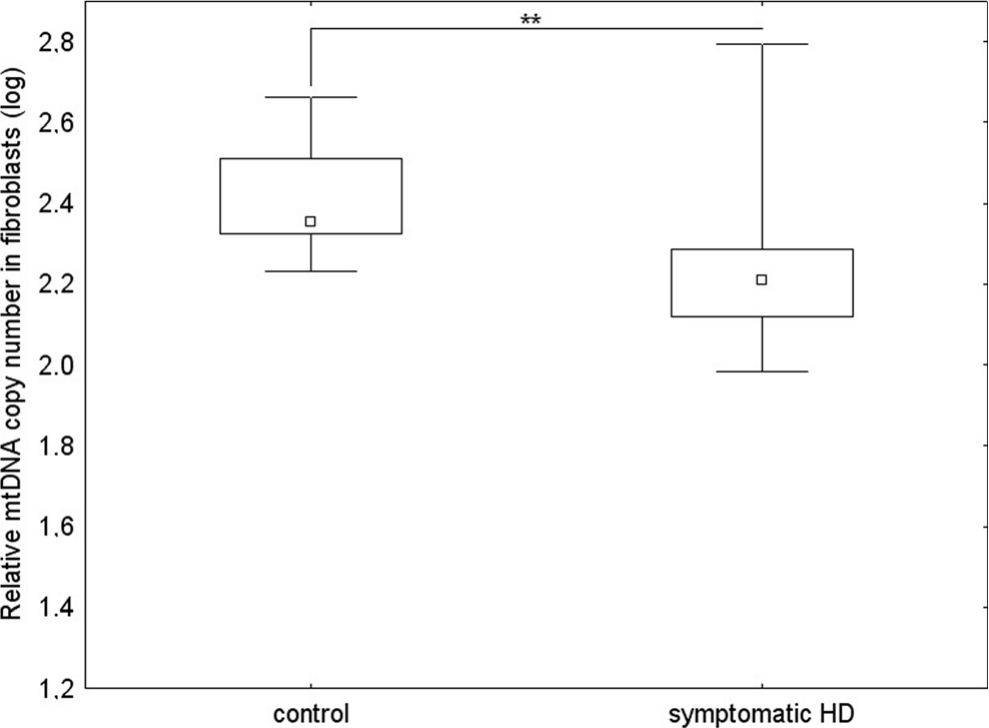
The relative mtDNA copy number in fibroblasts of healthy subjects and symptomatic HD patients, from whom blood samples and skin biopsies were taken, without division into sexes by the Mann-Whitney test on log-transformed data: *p < 0.05, ** *p* < 0.01, ***p < 0.001. The box and whisker plot represent median, 25% and 75% percentiles, and minimum and maximum

The differences between mtDNA/nDNA relative copy number in fibroblasts from healthy controls and symptomatic HD patients, without division into sexes, were assessed. The average mtDNA/nDNA relative copy number in fibroblasts was significantly lower in symptomatic HD patients compared to the controls (U Mann-Whitney test, *p* < 0.01) (Fig. 2).

**Fig. 3:**
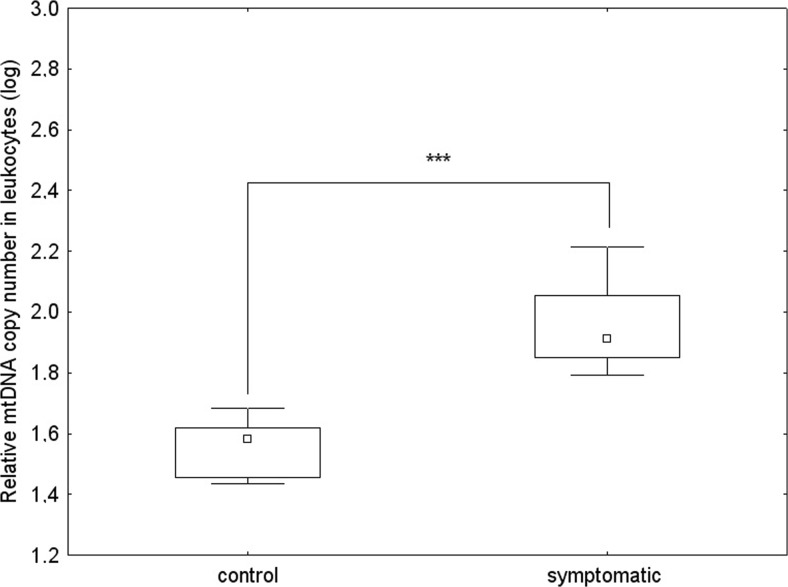
The relative mtDNA copy number in leukocytes of healthy subjects and symptomatic HD patients, from whom blood samples and skin biopsies were withdrawn, without division into sexes by the Mann-Whitney test on log-transformed data: *p < 0.05, ** *p* < 0.01, ***p < 0.001. The box and whisker plot represent median, 25% and 75% percentiles, and minimum and maximum

**Fig. 4:**
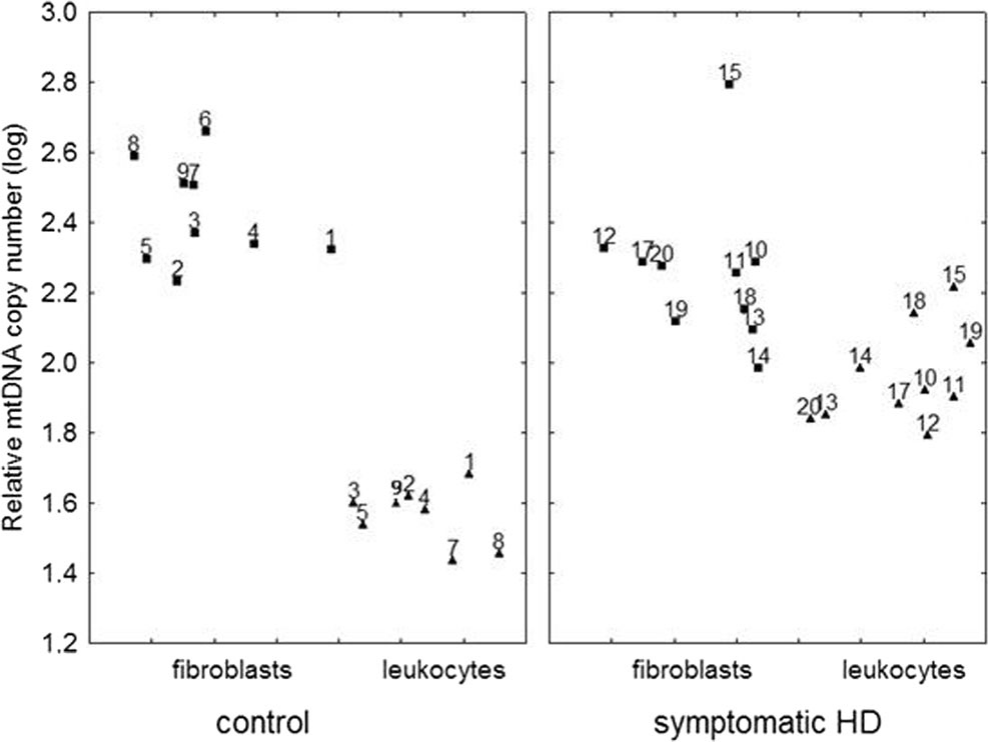
The comparison of the relative mtDNA copy number in fibroblasts and leukocytes of symptomatic HD patients and the control group. Triangles represent the mtDNA level in fibroblasts, and squares represent the mtDNA level in leukocytes, of the corresponding subjects (as numbered). The sample 6 of leukocytes of the control group was not analysed

Among subjects who donated both a blood sample and skin biopsies, we observed differences between the mtDNA/nDNA relative copy number in HD patients vs. controls measured in leukocytes and in fibroblasts. In contrast to fibroblasts, where mtDNA levels were lower relative to healthy subjects, the average mtDNA/nDNA relative copy number in leukocytes was significantly higher in symptomatic HD patients (Mann-Whitney test, *p* < 0.001), in accordance with the tendency observed for the whole groups investigated (Fig. 2 and Fig. 3). Therefore our results indicate that the differences in the mtDNA level between the groups (controls and HD patients) are going in opposite directions, depending on the examined tissue. This phenomenon is corroborated by an observation that although in the control group the mtDNA level was significantly increased in fibroblasts relative to leukocytes, this difference was almost absent in the case of symptomatic HD patients (as depicted in Fig. 4).

### Lack of association between mtDNA level and most disease severity measures

We aimed to correlate mtDNA levels in leukocytes with various factors, such as: age, number of CAG repeats, disease duration and factors influencing disease severity, such as: the Total Functional Capacity (TFC) score, Unified Huntington’s Disease Rating Scale (UHDRS) functional capacity, UHDRS total motor score, UHDRS oculomotor score, UHDRS maximal chorea score, depression score from Hospital Anxiety and Depression Scale, between presymptomatic HD patients and symptomatic HD patients with and without division into sexes. In symptomatic HD patients time since motor symptoms onset was also analysed. We found weak correlation between mtDNA level and time since motor symptoms onset but only without division into sexes (*r* = −0.2785, *p* < 0.05). No correlations between mtDNA/nDNA relative copy number in leukocytes and other disease parameters were found (details are provided in Supplementary Table S2). We also did not find any differences in mtDNA level depending on the sex of the parent from whom the mutated gene was inherited with and without division of the patients into sexes (data not shown). Height, weight, BMI (BodyMass Index), alcohol and cigarette smoking (number of units of alcohol drunk per week, number of cigarettes smoked per day and years of smoking) in presymptomatic and symptomatic HD patients with and without division into sexes also indicated no correlation with mtDNA level in leukocytes (data not shown). An analysis of covariance (ANCOVA) was carried out with mtDNA level entered as a dependent variable, sex, smoking status and alcohol abuse (the latter two classified as never, past or current) entered as fixed factors, selected UHDRS assessments (motor, functional), the TFC, disease burden (i.e. CAGn larger allele (Mengel-From et al. 2014), age at examination, disease duration and AOO) entered as covariates in all analyses. None of the analyses revealed statistically significant results. The analyses confirmed the influence of the sex on the mtDNA level (data not shown).

### Haplogroup analysis

Haplogroups were established for 79 healthy controls and 84 HD patients, both presymptomatic and symptomatic. Distribution of haplogroups among the analysed groups was similar (Table 4). We did not observe changes in frequency of any particular haplogroup among HD patients in comparison with the healthy controls. The haplogroup distribution was similar to that obtained in earlier studies for the Polish population (Malyarchuk et al. 2002; Piechota et al. 2004). No correlations between mtDNA/nDNA relative copy number in leukocytes and haplogroups were found.

**Table 4:** The distribution of haplogroups in the analysed groups of patients

Haplogroup	Symptomatic HD - number (%)	Presymptomatic HD - number (%)	Control - number (%)
V	0	0	0
H	18 (37.5)	6 (35.3)	22 (34.4)
U	10 (20.9)	4 (23.4)	14 (21.9)
X	0	0	1 (1.6)
Uk	3 (6.2)	1 (5.9)	5 (7.8)
J	4 (8.4)	1 (5.9)	7 (10.9)
T	8 (16.7)	0 (0)	9 (14)
R	1 (2.1)	2 (11.8)	0
I	0	0	1 (1.6)
D	2 (4.2)	0	0
C	0	2 (11.8)	0
F	1 (2)	0 (0)	4 (6.2)
A	1 (2)	0 (0)	0 (0)
Y	0	1 (5.9)	0 (0)
W	0	0	1 (1.6)
G	0	0	0
Total	48 (100)	17 (100)	64 (100)

### Common deletion analysis

**Fig. 5:**
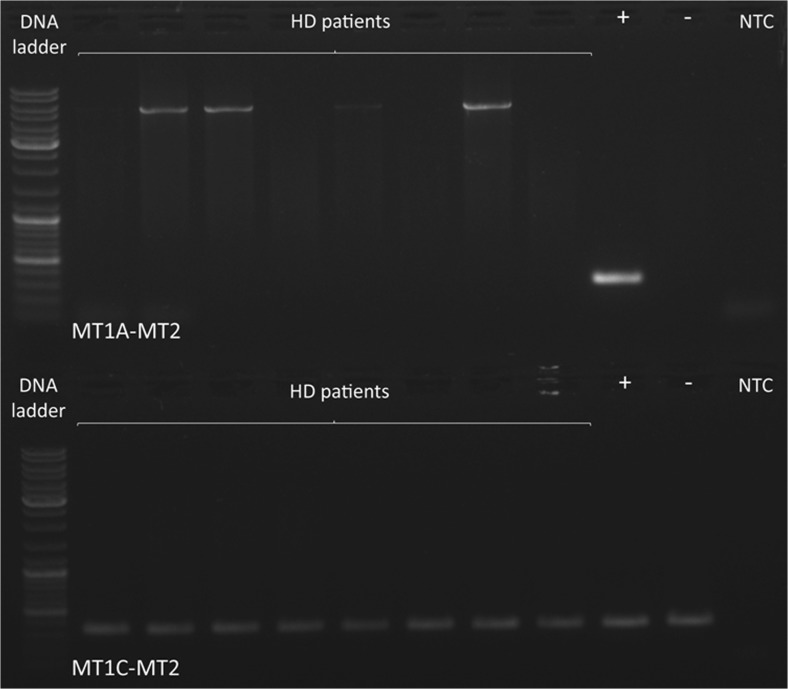
An exemplary electrophoregram of products obtained in the PCR detecting the presence of the common deletion. Primers MT1A-MT2 are complementary to the mtDNA sequence around breakpoints, and in the presence of the common deletion give a PCR product of approximately 300 bp. No product or, in some cases, only wild-type product (around 5 kb) indicates lack of the common deletion (upper panel). Primer MT1C is complementary to the region removed by the common deletion. Due to the fact that all of the wide range mtDNA deletions are heteroplasmic, PCR with primers MT1C-MT2 is used as a control reaction and should give a product of around 300 bp for every sample (lower panel). + − positive control with previously confirmed presence of common deletion, − - negative control without the common deletion, NTC- non template control

A 4977 bp deletion is the most common rearrangement in all human tissues and accounts for almost all cases of mtDNA deletions detected in blood. Therefore, as a routine method, PCR detecting the presence of the common deletion was performed to establish mtDNA quality and verify whether the patients’ mitochondrial genome stability was disturbed. We excluded the presence of the common deletion in all tested DNA samples. An exemplary electropherogram of products obtained in the PCR detecting the presence of the common deletion is presented in Fig. 5.

## Discussion

Several reports have been published indicating changes in the mtDNA amount in leukocytes of HD patients in comparison to healthy subjects (Chen et al. 2007; Liu et al. 2008; Petersen et al. 2014). Therefore, implications for the pathophysiology of HD could be suggested in relation to mtDNA level, however, different results were obtained in different laboratories. Therefore, a very important aspect of this study was to verify those contradictory findings, and just as importantly, to identify the potential sources of those discrepancies. In our study we analysed a large group of HD patients originating from Poland, a geographic region different from that analysed in the previous studies. We determined mtDNA levels in leukocytes of 62 symptomatic and 22 presymptomatic HD patients, and 79 age- and sex-matched healthy subjects. We found that symptomatic HD patients had a significantly higher mean level of mtDNA compared to healthy subjects. This observation corroborates the conclusions of Chen et al. (2007), but it is opposite to those of Liu et al. (2008), and Petersen et al. (2014). Moreover, we observed that among symptomatic HD patients, women had significantly higher mtDNA levels than men.

The observation that mtDNA levels differ between male and female HD patients is unexpected, and has never been published previously. It is worth mentioning that in our analyses no significant differences in mtDNA levels between healthy men and women were found. This is in accordance with previously published results demonstrating no sex-related differences in mtDNA copy number in peripheral blood cells, either in the crude or the age-adjusted analyses in healthy subjects (Mengel-From et al. 2014). The difference in mtDNA levels between the two sexes among HD patients led us to examine both sexes separately, searching for the cause of this difference. Both groups, men and women, had a similar course of the disease (AOO, CAG repeats, disease duration). It was reported previously that women displayed faster rates of HD progression than men (Zielonka et al. 2013). The observed differences were small, but statistically significant. These authors concluded that hormonal factors, differences in concomitant disorders, medication use, as well as genetic factors may contribute to the observed sex differences in disease severity. As sex can affect the mtDNA levels in HD patients’ blood cells, this type of analysis should be carried out taking the sex of the patient into consideration.

The increase in mtDNA copy number which is observed in the leukocytes of HD patients could be due to an attempt to compensate for whatever defect is caused by the presence of the mutated huntingtin protein. It was reported earlier that mtDNA amplification can be a compensatory mechanism in response to inefficient mitochondrial respiratory function (Bai et al. 2004) and such an increase has been observed in carriers of mutations in mitochondrial complex I genes which can lead to Leber’s Hereditary Optic Neuropathy (LHON) (Giordano et al. 2014). However, in LHON the amplification appears to affect the penetrance of the disease, as persons with the mutation but with a higher mitochondrial DNA copy number in blood, a tissue similarly unaffected as in HD, are healthy, whereas the persons who have lower amounts of mtDNA become blind. In this light, it is worth to note that different MHtt levels in isolated monocytes, T cells, and B cells (considered as different types of leukocytes) have been reported (Weiss et al. 2012). Moreover, an increase in the MHtt level in HD patients along with the presymptomatic or symptomatic status of the patient was observed. On the other hand, we have noted an increase in the amount of mtDNA (this work). Taking both observations into consideration, it might be possible that the increased mtDNA level can be a compensatory mechanism in response to higher level of MHtt.

There are several stages at which mtDNA replication could be upregulated, including transcription of mtDNA, protein transport through the mitochondrial membrane, dysregulation of expression of nuclear genes involved in mtDNA replication, and others. Assuming that MHtt affects the result of cellular functions at various stages, the dysregulation of the mtDNA amount might be due to several related reasons.

There are many factors that can affect mtDNA levels. All these factors might be potentially responsible for the differences between results published earlier by Chen et al. (2007), Liu et al. (2008) and Petersen et al. (2014). Malik and Czajka (2013) observed that the real time quantitative PCR method used to measure mtDNA levels may result in incorrect conclusions due to several problems connected with the methodology, including choice of mitochondrial and nuclear primers, a dilution bias or protocols used for template preparation. In those studies very small groups of patients and controls were investigated. Our results show that variance of mtDNA level in leukocytes of patients but also healthy persons is very high. Determination of the mtDNA level in the blood in a small number of patients or controls may therefore be insufficiently accurate. Therefore, in our study we analysed a large group of HD patients. According to our observations, the size of the analysed population also appears to be crucial. We proved this by statistical analysis of results taken from a reduced number of randomly selected samples. This resulted in comparison of the results presented in this report, obtained after analysis of the whole tested population, with those coming from the same population, but with the number of subjects comparable to that published earlier (Chen et al. 2007; Liu et al. 2008; Petersen et al. 2014). Contrary to a higher level of mtDNA in HD patients relative to the control group observed during the analysis of the population of 62 patients and 79 healthy persons, no such difference in the mtDNA level was found when the numbers of subjects were randomly decreased to those analysed by the Chen et al. (2007) and Liu et al. (2008), i.e. 36, 50, 40 and 16, 17, 18 subjects, respectively. Details of these statistical calculations are provided in Supplementary Table S3. The trend of continuing differences between men and women suffering from HD remained unchanged and was statistically significant, irrespective of the size of analysed population. Of course, we cannot exclude that the compared groups differ in respect to CAG expansion, disease severity, disease duration and other parameters. Apart from those potential differences, our results clearly show that the size of the analysed population has a significant influence on the obtained results. This factor, as well as others, mentioned above, may have direct impact on proper estimation of the association of mtDNA level with HD, which makes the results from different sources very difficult to compare.

Another aim of this study was to look for possible correlations between the mtDNA level and other measures from UHDRS. We have performed a comprehensive and multifactorial statistical analysis choosing the following parameters: age at blood donation, the CAG repeat expansion, the Total Functional Capacity (TFC) score, motor score, oculomotor score, maximal chorea score, depression score from HADS, time since motor symptoms onset, and Mini Mental State Examination (MMSE) and two specific cognitive scores (Symbol Digit Modalities Test, SDMT total correct - raw score, semantic fluency - animal names per minute, raw score). Most of these parameters (apart from MMSE and depression score) are quite sensitive to HD progression. However, none of these scores revealed correlation with mtDNA level in the case of symptomatic HD patients. We found a weak correlation between mtDNA level and time since motor symptoms onset: the longer the duration of motor symptoms, the lower the level of mtDNA, although this correlation concerned the analysis without division into sexes.

The opposite results obtained when mtDNA amounts were determined in leukocytes and dermal fibroblasts of the same patients are unexpected. We found a decreased mtDNA amount in fibroblasts of HD patients relative to the controls, while an increased mtDNA level was evident in leukocytes of the same group of patients when compared to healthy subjects. In our study, we also observed a significant difference in mtDNA levels between fibroblasts and leukocytes of subjects in the control group. In the case of symptomatic HD patients, the difference between mtDNA levels in fibroblasts and leukocytes was relatively small. We suggest that this phenomenon might be caused by medications taken by HD patients at the time of blood sample donation. Fibroblasts obtained from patients were first cultured under laboratory conditions, and only then the mtDNA levels were determined while mtDNA from leukocytes was examined shortly after sample withdrawal without culturing. In fact, mtDNA levels in fibroblasts of HD patients were significantly lower than those in healthy subjects, however, they were quite similar to those in leukocytes of both HD patients and control subjects. The specific mechanism of the differences remains unknown, but evidently, there are tissue specific variations in mtDNA levels in a single organism, which might be particularly pronounced when HD patients and control subjects are compared.

Another problem when analysing the obtained results is the possibility that medications aimed at motor and neuropsychiatric symptoms of HD patients might influence the mitochondrial functions. Patients analysed in this study, at the time of blood sample withdrawal, were taking medicines from the antipsychotic and antidepressant groups which have been found to mediate oxidative stress, inhibit the activity of mitochondrial complexes, influence mitochondrial functions or directly damage mitochondria, or mitochondrial DNA (Liu et al. 2003; Dean 2006; Boelsterli and Lim 2007; Atig et al. 2009; Finsterer et al. 2012; Siddiqui et al. 2012; Gómez et al. 2014). Moreover, HD patients received additional medications, which were not directly connected to this disease, but to other, unrelated and accompanying disorders, that, however, could also affect mitochondria. Some of the drugs (whose impact on mitochondria is confirmed in the literature) taken by HD patients analysed in this study, are listed in Supplementary Table S4. Therefore, we should bear in mind the possibility that mtDNA levels in the treated patients may be affected by the drugs. This makes a direct comparison of mtDNA levels between different patient cohorts in various studies difficult. Due to heterogeneity of medical treatment in HD patients, it is also almost impossible to collect uniform groups matched for demographics, sex, and pharmacological treatment. Thus, any comparison of results obtained for different groups of patients must be very careful, and perhaps direct conclusions cannot be drawn from such analyses.

## Conclusion

In our study, an increased mtDNA/nDNA copy number in blood leukocytes, and a decreased mtDNA/nDNA copy number in dermal fibroblasts derived from HD patients (both presymptomatic and symptomatic) relative to the control group was observed. HD women displayed a higher level of mtDNA in leukocytes than HD men. The type of haplogroup of patients had no influence on the mtDNA level in cells. We did not observe the common deletion in mtDNA of HD patients. We conclude that the type of analysed tissue and the number of analysed subjects should be considered when mtDNA level is determined.

## Electronic supplementary material

ESM 1 (PDF 529 kb)
