# Supplementary material for: Mitochondrial DNA levels in Huntington disease leukocytes and dermal fibroblasts
Source: Metab Brain Dis. 2017 May 16;32(4):1237–47. doi: 10.1007/s11011-017-0026-0 (PMC5504138; doi:10.1007/s11011-017-0026-0)
Supplement: Supplementary file 1 — (PDF 529 kb) [file 11011_2017_26_MOESM1_ESM.pdf]

Mitochondrial DNA levels in Huntington disease leukocytes and fibroblasts

Paulina Jędrak[1], Magdalena Krygier[2], Katarzyna Tońska[3], Małgorzata Drozd[3], Magdalena Kaliszewska[3], Ewa Bartnik[3,4], Witold Sołtan[5,6], Emilia J. Sitek[5,6], Anna Stanisławska-Sachadyn[2], Janusz Limon[2], Jarosław Sławek[5,6], Grzegorz Węgrzyn[1], Sylwia Barańska[1]*

[1] Department of Molecular Biology, University of Gdańsk, Poland
[2] Department of Biology and Genetics, Medical University of Gdańsk, Poland

[3] Institute of Genetics and Biotechnology, Faculty of Biology, University of Warsaw , Poland

[4] Institute of Biochemistry and Biophysics, Polish Academy of Sciences, Warsaw, Poland

[5] Department of Neurological and Psychiatric Nursing, Medical University of Gdańsk, Poland
[6] Department of Neurology, St. Adalbert Hospital, Copernicus PL Ltd., Gdansk, Poland

*** Corresponding author:**

Dr. Sylwia Barańska

Department of Molecular Biology, University of Gdańsk, Wita Stwosza 59 , 80-308 Gdańsk, Poland

e-mail: sylwia.baranska@biol.ug.edu.pl

Supplementary Table S1. Primers used in qPCR.

Oligonucleotide name	Sequence (5'-3')	Target/Gene	Product size (bp)	Annealing temp.
16S F	cgaaaggacaagagaaataagg	mtDNA/16S	152	60°C
16S R	ctgtaaagttttaagttttatgcg	mtDNA/16S		
ß-globin F	caacttcatccacgttcacc	nDNA/ß-globin	268	53°C
ß-globin R	gaagagccaaggacaggtac	nDNA/ß-globin		

Supplementary Table S2. Data correlation analysis of the relationship between mtDNA level and the selected UHDRS assessments of HD patients. Correlation coefficients with the respective p value are displayed. Statistically significant values are marked by asterisks
N - no scores due to low number of subjects with the assessed parameter, **UHDRS**- Unified Huntington's Disease Rating Scale, **HADS-SIS**- Hospital Anxiety and Depression Scale combined with the Snaith Irritability Scale, **MMSE** – Mini Mental State Examination, **SDMT** - Symbol Digit Modalities Test total correct,

Parameter	symptomatic HD patients						presymptomatic HD patients					
	both sexes		women		men		both sexes		women		men	
	r	p	r	p	r	p	r	p	r	p	r	p
Age (years)	0.0034	0.979	0.1572	0.353	0.0224	0.916	-0.3159	0.152	-0.4774	0.053	0.7626	0.177
Disease duration (years)	-0.1305	0.312	0.0816	0.626	0.1785	0.404	N	N	N	N	N	N
CAG repeat expansion	0.0393	0.762	0.163	0.335	0.1792	0.391	0.0762	0.736	0.1323	0.613	-0.4141	0.488
the Total Functional Capacity (TFC) score	-0.0011	0.993	0.1318	0.432	0.21	0.573	N	N	N	N	N	N
UHDRS functional capacity	-0.0258	0.8829	0.2877	0.2799	-0.2976	0.2159	N	N	N	N	N	N
depression score from HADS-SIS	-0.1148	0.4926	-0.0485	0.8345	-0.0568	0.8287	0.0414	0.858	0.0383	0.888	0.1943	0.754
UHDRS maximal chorea score	-0.1382	0.2839	-0.0277	0.8709	-0.3768	0.0634	N	N	N	N	N	N
UHDRS total motor score	-0.0423	0.746	-0.0524	0.758	-0.1133	0.598	N	N	N	N	N	N
UHDRS oculomotor score	0.0423	0.746	0.0225	0.895	0.114	0.596	N	N	N	N	N	N
UHDRS time since motor onset	-0.2785*	0.03*	-0.2295	0.172	-0.266	0.209	N	N	N	N	N	N
Cognitive – SDMT	0.0319	0.821	0.0233	0.904	-0.1628	0.447	N	N	N	N	N	N
Cognitive - semantic fluency raw score (animal names / min.)	0.0986	0.457	0.0856	0.620	0.1326	0.546	N	N	N	N	N	N
MMSE raw score	-0.1089	>0.05	0.0750	>0.05	-0.3894	>0.05	N	N	N	N	N	N

Supplementary Table S3. Results of statistical calculations obtained by Chen *et al.* (2007), Liu *et al.* (2008) and Petersen *et al*. (2014) and in this study. Calculation results obtained after matching the number of subjects tested in this work to that analyzed by Chen *et al*. (2007), Liu *et al*. (2008) or Petersen *et al*. (2014) are also included.

We aimed to check whether the population size can influence the results of analyses of differences between mtDNA levels in HD patients and the control group. First, because of the difference in the size of male and female groups, we decided to match the number of subjects in both groups. In order to achieve this, the population of female HD patients in our database was randomized using the software Statistica 10 and decreased in size to get equal numbers in both groups, men and women. In this way, we formed 3 randomized sets. Data prepared in this way were used to determine the mean value of mtDNA levels in both groups and to compare these levels between groups. Each analysis demonstrated a statistically significant higher level of mtDNA in female HD patients relative to male patients. Therefore, a decrease in the size of population of female patients did not change the general trend revealed for the whole population of HD patients.

We then performed another analysis, by decreasing the number of subjects tested in this study to that analyzed by Chen *et al.* (2007), Liu *et al*. (2008) or Petersen *et al.* (2014). For this purpose, from 79 control and 62 HD patients, we randomly selected groups of 36 (control) and 16 (HD) subjects according to Chen *et al.* (2007), 50 (control) and 17 (HD) subjects according to Liu *et al.* (2008), and 28 (control) and 41 (HD) subjects according to Petersen *et al*. (2014). In each case, randomizing was performed three times, in order to obtain three separate sets of data, which were used to determine independently the mean value of the mtDNA level. In the case of lower numbers of analyzed subjects, i.e. according to the reports by Liu *et al*. (2008) and Chen *et al.* (2007), from the three analyses performed, one revealed a statistically significant increase in the mtDNA level in HD patients relative to controls, and two showed no significant differences in mtDNA levels between HD patients and healthy subjects. In the case of the analysis of subjects equal in number to that described by Petersen *et al*. (2014), our results have shown a statistically significant increase of mtDNA level in HD patients relative to the control group in each set. Thus, on the basis of these statistical considerations, we believe that the population size is of great importance in such analyses.

Supplementary Table S3

Report	sets	number of subjects		The mtDNA copy number in leukocytes in HD patients compared to control
		n_1=control	n_2=HD patients	
Chen *et al.,* 2007		n_1= 36	n_2= 16	increase (p=0.046)
Liu *et al.,* 2008		n_1= 50	n_2= 17	decrease (p=0.037)
Petersen *et al.*, 2014		n_1= 28	n_2= 41	decrease (p<0.0001)
This work		n_1= 79	n_2= 62	increase (p<0.001)
This work with 3 sets of randomized study group to match the size of the study groups of Chen *et al*., 2007 and Liu *et al*., 2008	set 1	n_1=36	n_2=16,	Equal
	set 2	n_1'= 50	n_2'=17	Equal
	set 3	n_1''= 40	n_2''= 18	increase (p<0.001)
This work with 3 sets of randomized study group to match the size of the study group of Petersen *et al.,* 2014	set 1	n_1=28	n_2=41	increase (p<0.001)
	set 2			increase (p<0.001)
	set 3			increase (p<0.001)

Supplementary TableS4. Selected drugs taken by HD patients analyzed in this study, whose impact on mitochondria is confirmed in the literature.

**Drug type**	**Drugs**	**Literature**
**Antipsychotics**	Clozapine, Haloperidol, Olanzapine, Quetiapine, Risperidone, Sulpride	Elmorsy and Smith, 2015; Burkhardt *et al.*, 1993; Dean, 2006; Gellerich and Zierz, 1997; Balijepalli *et al.*, 1999, Modica-Napolitano *et al.*, 2003; Casademont *et al.*, 2007; Vucicevic *et al.*, 2014; Fisar *et al.*, 2010; Garabadu *et al.*, 2015
**Antidepressants**	Bupropion, Buspirone, Citalopram, Doxepin, Mirtazapine, Paroxetine, Sertraline, Trazodone, Venlafaxine	Woynillowicz *et al.*, 2012; Dykens *et al.*, 2008; Fisar *et al.*, 2010; Xia *et al.*, 1999; Roth, 1975; Garabadu *et al.*, 2015; Li *et al.*, 2012
**Anticonvulsants**	Carbamazepine, Clonazepam, Valproic acid,	Santos *et al.*, 2008; Consolini *et al.*, 2007; Jafarian *et al.*, 2013; Hass *et al.,* 1981; Rumbach *et al.,* 1983; *Luís et al.*, 2007; Finsterer, 2012
**Anxiolytic and sedative drugs**	Buspirone, Hydroxyzine	Dykens *et al.*, 2008; Nishi *et al.*, 1959
**Statins**	Atorvastatin	Singh *et al.*, 2015; Kucharska *et al.*, 2010; Abdoli *et al.*, 2013
**Angiotensin-converting-enzyme (ACE) inhibitor**	Ramipryl, Enalapril, Quinapril	Ulicná *et al.,* 1997; Marzetti *et al.*, 2013; Inserra *et al.*, 1995; Ferder *et al.*, 2002; de Cavanagh *et al.*, 2003; de Cavanagh *et al.*, 2008; Sato *et al.*, 2003

**Angiotensin II receptor antagonist**	Candesartan	Gaur and Kumar, 2011
**Beta-blockers**	Atenolol, Metoprolol, Bisoprolol, Nebivolol	Gómez *et al.*, 2014; Sharma and McNeill, 2011; Ichihara *et al.*, 2006; Huang *et al.*, 2013; Khan *et al.,* 2013
**Angiotensin II type 1 receptor (AT_1) antagonists**	Losartan	de Cavanagh *et al.*, 2008; 2003
**A thiazide-like diuretic**	Indapamid	Mouhieddine *et al.,* 1993
**Long-acting dihydropyridine-type calcium channel blockers**	Amlodipine	Mamou *et al.*, 2015
**L-type calcium channel blocker**	Verapamil	Yu *et al.*, 2014
**Salicylate drug**	Acetylsalicylic acid (Aspirin, ASA)	Raza and John, 2012; Somasundaram *et al.*, 1997
**Proton pump inhibitor**	Omeprazole	Tonazzi *et al.*, 2013
**Diabetes medicine**	Metformin	Viollet *et al.,* 2012

References:

Abdoli N, Heidari R, Azarmi Y & Eghbal MA (2013) Mechanisms of the statins cytotoxicity in freshly isolated rat hepatocytes. J. Biochem. Mol. Toxicol. 27: 287-294. doi:10.1002/jbt.21485..

Balijepalli S, Boyd MR & Ravindranath V (1999) Inhibition of mitochondrial complex I by haloperidol: The role of thiol oxidation. Neuropharmacology 38: 567–577. doi:10.1016/S0028-3908(98)00215-9.

Burkhardt C, Kelly JP, Lim YH, Filley CM & Parker WD (1993) Neuroleptic medications inhibit complex I of the electron transport chain. Ann. Neurol. 33: 512-517. doi:10.1002/ana.410330516.

Casademont J *et al.* (2007) Neuroleptic treatment effect on mitochondrial electron transport chain: peripheral blood mononuclear cells analysis in psychotic patients. J. Clin. Psychopharmacol. 27: 284-288. doi: 10.1097/JCP.0b013e318054753e.

Chen CM *et al.* (2007) Increased oxidative damage and mitochondrial abnormalities in the peripheral blood of Huntington's disease patients. Biochem. Biophys. Res. Commun. **359:** 335–340. doi:10.1016/j.bbrc.2007.05.093.

Consolini AE, Ragone MI, Conforti P & Volonté MG(2007) Mitochondrial role in ischemia-reperfusion of rat hearts exposed to high-K+ cardioplegia and clonazepam: energetic and contractile consequences. Can. J. Physiol. Pharmacol. 85: 483-496. doi:10.1139/Y07-022.

De Cavanagh EMV, Flores I, Ferder M, Inserra F & Ferder L (2008) Renin-angiotensin system inhibitors protect against age-related changes in rat liver mitochondrial DNA content and gene expression. Exp. Gerontol. 43: 919–928. doi:10.1016/j.exger.2008.08.007.

De Cavanagh EMV (2003) Enalapril and losartan attenuate mitochondrial dysfunction in aged rats. FASEB J. 17: 1096–1098. doi:10.1096/fj.02-0063fje.

Dean CE (2006) Antipsychotic-associated neuronal changes in the brain: Toxic, therapeutic, or irrelevant to the long-term outcome of schizophrenia? Prog. Neuro-Psychopharmacol. Biol. Psychiatry 30: 174–189. doi:10.1016/j.pnpbp.2005.08.019

Dykens JA *et al.* (2008) In vitro assessment of mitochondrial dysfunction and cytotoxicity of nefazodone, trazodone, and buspirone. Toxicol. Sci. 103: 335–345. doi:10.1093/toxsci/kfn056

Elmorsy E & Smith PA (2015) Bioenergetic disruption of human micro-vascular endothelial cells by antipsychotics. Biochem. Biophys. Res. Commun. 460: 857–862. doi:10.1016/j.bbrc.2015.03.122

Ferder LF, Inserra F & Basso N (2002) Advances in our understanding of aging: Role of the renin-angiotensin system. Curr. Opin. Pharmacol. 2: 189–194. doi:10.1016/S1471-4892(02)00139-X

Finsterer J (2012) Mitochondrion-toxic drugs given to patients with mitochondrial psychoses. Behav. Brain Funct. 8: 45. doi:10.1186/1744-9081-8-45.

Fišar Z, Hroudová J & Raboch J (2010) Inhibition of monoamine oxidase activity by antidepressants and mood stabilizers. Neuroendocrinol. Lett. 31: 645

Garabadu D, Ahmad A & Krishnamurthy S (2015) Risperidone Attenuates Modified Stress–Re-stress Paradigm-Induced Mitochondrial Dysfunction and Apoptosis in Rats Exhibiting Post-traumatic Stress Disorder-Like Symptoms. J. Mol. Neurosci. 56: 299–312. doi:10.1007/s12031-015-0532-7.

Gaur V & Kumar A (2011) Neuroprotective potentials of candesartan, atorvastatin and their combination against stroke induced motor dysfunction. Inflammopharmacology 19: 205–214. doi:10.1007/s10787-010-0068-y

Gellerich FN & Zierz SM (1997) Detection of Mitochondrial Diseases (ed. Gellerich, F.N. & Zierz) 249-259. doi:10.1007/978-1-4615-6111-8. (Springer Science & Business Media 1997).

Gómez A. *et al.*(2014) Lifelong treatment with atenolol decreases membrane fatty acid unsaturation and oxidative stress in heart and skeletal muscle mitochondria and improves immunity and behavior, without changing mice longevity. Aging Cell 13: 551–560. doi:10.1111/acel.12205

Haas R, Chir B, Stumpf DA, Parks JK & Eguren L (1981) Inhibitory effects of sodium valproate on oxidative phosphorylation. Neurology 31: 1473-1473.

Huang C, Chen D, Xie Q, Yang Y. & Shen W (2013) Nebivolol stimulates mitochondrial biogenesis in 3T3-L1 adipocytes. Biochem. Biophys. Res. Commun. 438: 211–217. doi:10.1016/j.bbrc.2013.07.055

Ichihara S *et al.* (2006) Attenuation of oxidative stress and cardiac dysfunction by bisoprolol in an animal model of dilated cardiomyopathy. Biochem. Biophys. Res. Commun. 350: 105–113. doi:10.1016/j.bbrc.2006.09.026.

Inserra F, Romano L, Ercole L, de Cavanagh EM & Ferder L (1995) Cardiovascular changes by long-term inhibition of the renin-angiotensin system in aging. Hypertension 25: 437-442 doi:10.1161/01.HYP.25.3.437

Jafarian I, Eskandari MR, Mashayekhi V, Ahadpour M & Hosseini MJ (2013) Toxicity of valproic acid in isolated rat liver mitochondria. Toxicol. Mech. Meth. 23: 617-623. doi:10.3109/15376516.2013.821567

Khan MU *et al.*(2013) Nebivolol: a multifaceted antioxidant and cardioprotectant in hypertensive heart disease. J. Cardiovasc. Pharm. 62: 445-451. doi: 10.1097/FJC.0b013e3182a0b5ff

Kucharska J *et al*.(2010) Effects of atorvastatin on heart mitochondrial function and coenzyme Q content in the experiment. Bratisl. lek. listy 112: 603-604.

Li Y, Couch L, Higuchi M, Fang J-L & Guo L (2012) Mitochondrial dysfunction induced by sertraline, an antidepressant agent. Toxicological Sciences 127: 582–591. doi: 10.1093/toxsci/kfs100.

Liu CS *et al.* (2008) Depletion of mitochondrial DNA in leukocytes of patients with poly-Q diseases. J. Neurol. Sci. 264**:** 18–21. doi:10.1016/j.jns.2007.07.016

Luís PBM (2007) *et al.* Valproic acid metabolites inhibit dihydrolipoyl dehydrogenase activity leading to impaired 2-oxoglutarate-driven oxidative phosphorylation. Biochim. Biophys. Acta - Bioenerg. 1767: 1126–1133. doi:10.1016/j.bbabio.2007.06.007.

Mamou Z *et al.* (2015) Effects of amlodipine and perindoprilate on the structure and function of mitochondria in ventricular cardiomyocytes during ischemia-reperfusion in the pig. Fundam. Clin. Pharmacol. 29: 21–30. doi:10.1111/fcp.12070

Marzetti E *et al*. (2013) Late-life Enalapril administration induces nitric oxide-dependent and independent metabolic adaptations in the rat skeletal muscle. Age (Omaha) 35: 1061–1075. doi:10.1007/s11357-012-9428-4.

Modica-Napolitano JS, Lagace CJ, Brennan WA & Aprille JR (2003) Differential effects of typical and atypical neuroleptics on mitochondrial function in vitro. Arch. Pharm. Res. 26: 951–959.

Mouhieddine S, Tresallet N, Boucher F & de Leiris J (1993) Ultrastructural basis of the free-radical scavenging effect of indapamide in experimental myocardial ischemia and reperfusion. J. Cardiovasc. Pharm 22: 47-52

Nishi S, Koketsu K, Cerf JA & Abood LG (1959) Some electrophysiological and biochemical studies with hydroxyzine. J. *Pharmacol. Exp. Ther*. 126: 148-154

Petersen MH *et al.* (2014) Reduction in mitochondrial DNA copy number in peripheral leukocytes after onset of Huntington's disease. Mitochondrion 17: 14–21. doi:10.1016/j.mito.2014.05.001.

Raza H & John A (2012) Implications of altered glutathione metabolism in aspirin-induced oxidative stress and mitochondrial dysfunction in HepG2 cells. PLoS One 7: doi:10.1371/journal.pone.0036325.

Roth JA (1975) Inhibition of rabbit monoamine oxidase by doxepin and related drugs. Life sciences 16: 1309-1319.

Rumbach L *et al.* (1983) Inhibition of oxidative phosphorylation in hepatic and cerebral mitochondria of sodium valproate-treated rats J. Neurol. Sci. 61: 417-423.

Santos NAG *et al.* (2008) Aromatic antiepileptic drugs and mitochondrial toxicity: Effects on mitochondria isolated from rat liver. Toxicol. Vitr. 22: 1143–1152. doi:10.1016/j.tiv.2008.03.004.

Sato H, Yaoita H, Maehara K & Maruyama Y (2003) Attenuation of heart failure due to coronary stenosis by ACE inhibitor and angiotensin receptor blocker. Am. J. Physiol. Heart Circ. Physiol. 285: H359–H368

Sharma V & McNeill JH (2011) Parallel effects of β-adrenoceptor blockade on cardiac function and fatty acid oxidation in the diabetic heart: Confronting the maze. World J. cardiol. 3: 281. doi:10.4330/wjc.v3.i9.281.

Singh F *et al.* (2015) Reductive stress impairs myoblasts mitochondrial function and triggers mitochondrial hormesis. Biochim. Biophys. Acta - Mol. Cell Res. 1853: 1574–1585. doi:10.1016/j.bbamcr.2015.03.006.

Somasundaram S *et al.* (1997) Mitochondrial damage: a possible mechanism of the “topical” phase of NSAID induced injury to the rat intestine. Gut 41: 344–353. doi:10.1136/gut.41.3.344.

Tonazzi A, Eberini I & Indiveri C (2013) Molecular mechanism of inhibition of the mitochondrial carnitine/ acylcarnitine transporter by omeprazole revealed by proteoliposome assay, mutagenesis and bioinformatics. PLoS One 8: 1–9. doi:10.1371/journal.pone.0082286.

Ulicna O *et al.* (1997) Bioenergetics of liver mitochondria after administration of ramipril in experimental diabetes mellitus. Bratisl. lek. listy 98: 687-694.

Viollet B *et al.* (2012) Cellular and molecular mechanisms of metformin: an overview. Clin. Sci. 122**:** 253-270

Vucicevic L *et al.* (2014) Autophagy inhibition uncovers the neurotoxic action of the antipsychotic drug olanzapine. Autophagy 10: 2362-2378. doi**:**10.4161/15548627.2014.984270.

Woynillowicz AK, Raha S, Nicholson CJ & Holloway AC (2012) The effect of smoking cessation pharmacotherapies on pancreatic beta cell function. Toxicol. Appl. Pharmacol. 265: 122–127. doi:10.1016/j.taap.2012.08.020.

Xia Z, Lundgren B, Bergstrand A, DePierre JW & Nässberger L (1999) Changes in the generation of reactive oxygen species and in mitochondrial membrane potential during apoptosis induced by the antidepressants imipramine, clomipramine, and citalopram and the effects on these changes by Bcl-2 and Bcl-X(L). Biochem. Pharmacol. 57**:** 1199–1208. doi:10.1016/S0006-2952(99)00009-X.

Yu Q *et al.* (2014) The Calcium Channel Blocker Verapamil Inhibits Oxidative Stress Response in Candida albicans. Mycopathologia 177: 167–177. doi:10.1007/s11046-014-9735-7.
